# Improving health-promoting workplaces through interdisciplinary approaches. The example of WISEWORK-C, a cluster of five work and health projects within Horizon-Europe

**DOI:** 10.5271/sjweh.4238

**Published:** 2025-07-01

**Authors:** Deborah De Moortel, Michelle C. Turner, Ella Arensman, Alex Binh Vinh Duc Nguyen, Víctor Gonzalez

**Affiliations:** 1 Compassionate Communities Centre of Expertise (COCO), Vrije Universiteit Brussel, Brussels, Belgium; 2 Brussels Institute for Social and Population Studies (BRISPO), Vrije Universiteit Brussel, Brussels, Belgium; 1 Barcelona Institute for Global Health (ISGlobal), Barcelona, Spain; 2 Universitat Pompeu Fabra (UPF), Barcelona, Spain; 3 CIBER Epidemiología y Salud Pública (CIBERESP), Madrid, Spain; 1 Head of School of Public Health, Professor of Public Mental Health, School of Public Health, College of Medicine and Health, Cork, Ireland; 2 Chief Scientist, National Suicide Research Foundation, University College Cork, Cork, Ireland; 3 EU Horizon Europe PROSPERH Consortium; 1 Research(x)Design, Department of Architecture, Faculty of Engineering Science, KU Leuven, Belgium; 1 SINTEF Digital, Department of Health Research, Oslo, Norway

Digitalization, green transitions, and demographic change are transforming societies and economies across Europe. These shifts are giving rise to new forms of work (eg, hybrid work, gig economy jobs) and reshaping management and work organization practices (eg, through algorithmic decision-making or digital monitoring of worker performance). While such developments offer important opportunities to improve sustainability, flexibility, and efficiency, they also present challenges for ensuring healthy and equitable working conditions—especially if workplace policies and practices do not keep pace with these transformations (1).

Work-related illnesses and injuries already place a substantial burden on employers and the broader economy, with costs estimated to exceed 3.3% of the European Union (EU) gross domestic product annually (2). It is well established that the work environment plays a crucial role in shaping both physical and mental health. Poorly designed or managed workplaces are associated with increased risks of musculoskeletal disorders (MSD), stress, burnout, and long-term sickness absence (3). In contrast, supportive work environments—characterized by ergonomic design, good environmental quality, and worker autonomy—have been shown to improve well-being, productivity, and job satisfaction (4).

Rapid advances in digital technologies, particularly artificial intelligence (AI), further amplify both opportunities and risks in the modern workplace. AI can help enhance safety, reduce physical demands, and streamline tasks, but also raises concerns about autonomy, fairness, transparency, and mental well-being (5). Understanding how these technologies reshape power dynamics, management practices, and psychosocial work environments is essential to ensuring responsible, inclusive, and health-promoting digital transitions.

Amid these transitions (6), MSD continue to be among the most common work-related health issues (7), while stress, depression, and anxiety are frequently cited by workers and managers as critical mental health concerns (8). The emergence of the COVID-19 pandemic has accelerated these changes, introducing additional mental health challenges and intensifying pre-existing physical risk factors (9). For instance, the increase in computer use, shift to non-traditional workspaces (eg, home offices), and reduced physical activity among office workers often result in prolonged static postures and repetitive movements, factors that elevate the risk of negative health outcomes (10). These post-pandemic trends are also linked to a rise in mental health issues such as stress, burnout, and social isolation (11).

In response to these challenges, the EU has launched several strategic initiatives aimed at ensuring safe, healthy, and inclusive working conditions. The EU Strategic Framework on Health and Safety at Work 2021–2027 calls for adapting occupational safety and health (OSH) practices to the realities of digitalization, demographic shifts, and new forms of work (12). Complementing this, the European Commission’s 2023 Communication on a comprehensive approach to mental health (13) sets mental health on equal footing with physical health, announcing 20 flagship actions backed by over €1.2 billion in funding. Among these are initiatives that specifically target psychosocial risks at work, including the development of an EU-level initiative on managing psychosocial risks (14) and the organization of EU-wide workplace campaigns to raise awareness and promote preventive action (15). These efforts align with the European Pillar of Social Rights (16), reinforcing the EU’s commitment to fair working conditions, universal access to healthcare, and robust social protections.

In this context, **WISEWORK-C** (Workplace Innovation for Sustainable Well-being Cluster) (www.wisework-c.eu) is a recently established cluster composed of five independent projects funded by the EU Framework Programme for Research and Innovation Horizon Europe under the call “HORIZON-HLTH-2023-ENVHLTH-02-02: Evidence-based interventions for the promotion of mental and physical health in changing working environments (post-pandemic workplaces)”. The cluster’s primary aim is to enhance collaboration and jointly promote the benefits of addressing mental and physical health in the workplace and develop and evaluate a range of evidence-based interventions. Using scientific findings, the cluster seeks to inform and influence policy and decision-makers to take greater action, ultimately leading to more sustainable, health-promoting workplaces.

To achieve these goals, WISEWORK-C brings together five Horizon Europe projects (see table 1) with unique interdisciplinary approaches:

**Table 1:** Comparison of five projects in WISEWORK-C. [JEM=job exposure matrices; cRCT=cluster randomized controlled trial]

Project	Context	Methods	Sectors and countries	Trial registration	Key stakeholders	Key outcomes
EU-CoWork	New ways of working might benefit or harm the health and well-being of employees faced with serious illness, loss or caregiving and their colleagues	Co-creative and developmental evaluation of 'compassionate workplace programs' and mixed-methods (meta-)process and impact evaluation	Primary, secondary and tertiary sectors (Austria, Belgium, Greece, Sweden)	Not applicable, no trial design	Employees, workplaces, unions and labor organizations, researchers, policy-makers and regulators society.	Reducing health-related suffering and maintaining or improving the well-being of workers with end-of-life experiences and their colleagues, evidence base for compassionate workplace programs
INTERCAMBIO	Climate change and the green transition	Intervention with co-creation and natural experiment	Outdoor construction, healthcare, public transit, wind turbine manufacturing, and waste management/recycling (Spain, The Netherlands, Switzerland, Denmark, Portugal, Sweden)	ISRCTN12209427, ISRCTN25033513, NCT06688721, ISRCTN10031201, ISRCTN19236285	Scientific community and other multi-level stakeholders including of industry partners, workers/unions, non-governmental organizations and policy-makers	Evidence-based recommendations; exposure measurement database; new quantitative JEM; policy framework; new health research agenda
PROSPERH	Impact of rapid changes in workplaces on physical and mental health of employees	Gather robust evidence on mental and physical health in the workplace; develop and validate (via a cRCT in 11 countries) the multi-level PROSPERH digital intervention	Workplaces, targeting healthcare, construction and telework and ICT based mobile work settings Implementation countries: Albania, Denmark, Ireland, Portugal, The Netherlands, Turkey, Germany, Australia, Spain, Hungary, Kosovo	Clinical study registration to be performed	Scientific community, policy makers (international, European and national), occupational health and safety professionals, industry, workplaces and employees	Evidence-based multi-level physical and mental health workplace intervention; policy recommendations; robust evidence published from literature review and analysis of high-quality datasets
SONATA	Unclear impact of architectural adaptive technologies on worker health and well-being.	Empirical evaluation of the individual and orchestrated impact of architectural adaptations on the worker health and wellbeing indicators	Three shared workplace contexts (public workplace, semi-public workplace, private workplace) across Belgium, Germany, Italy, and The Netherlands	Clinical study registration under processing.	Architects, designers, adaptive technologies manufacturers, workplace managers, policymakers, workers	Evidence-based recommendations on how architectural adaptations can actively benefit human health and wellbeing in shared workplace contexts
WAge	Ageing populations and extended working lives raise challenges for maintaining health and work ability. Integrated, age-sensitive approaches to workplace health remain limited	Mixed-methods design combining observational and sensor-based physical exposure assessments with self-reported psychosocial and health data; participatory research; integrative modelling	Manufacturing and services sectors (Poland, Portugal, Spain)	Clinical study registration under processing	Workers, companies, unions, occupational health professionals, policy makers, researchers	WAge Index – an integrative assessment and recommendation tool for targeted interventions; participatory governance model for workplace health research; policy and intervention guidelines

(i) **EU-CoWork.** EU-CoWork’s ambition is to develop an evidence basis for and subsequently develop, test and evaluate tailored 'compassionate workplace programs' (17) in Europe to leverage the positive impacts and existing assets of workplaces and mitigate the negative impacts and challenges of the new ways of working for the mental and physical health and well-being of employees faced with end-of-life experiences (such as loss, grief, dying, death, serious illness and care giving) and their colleagues (18). The project is a collaboration between five countries (Austria, Belgium, Greece, the UK, and Sweden) and entails a cross-national mixed-methods intervention study with an embedded process and impact evaluation. It applies an international co-creative and developmental evaluation of tailored compassionate workplace programs, and mixed-methods process and impact evaluation combining a timed series of quantitative cross-sectional panel surveys, qualitative interviews and fieldwork, and policy document analysis. Tailored compassionate workplace programs will be developed in 12 digitalized and/or green workplaces across four European countries.

(ii) **INTERCAMBIO.** There has been insufficient attention to the impacts of climate change and the green transition on occupational health and safety (19). The INTERCAMBIO project aims to promote the health of workers in work environments impacted by climate change, implementation of new sustainable work practices, and the green transition (20). The project is a collaboration between 14 partners in eight countries (Belgium, Denmark, The Netherlands, Portugal, Spain, Sweden, Switzerland, and the UK). INTERCAMBIO addresses mental and physical health of workers through specific workplace interventions in five relevant industries including outdoor construction, healthcare, public transit, wind turbine manufacturing, and waste management/recycling using mixed-methods. In addition, INTERCAMBIO is also performing observational research to evaluate both short- and long-term impacts of occupational heat, cold, and solar UV radiation exposures in relation to mental and physical health effects, including in biomarker-based studies, as well as in large-scale cohort studies using innovative exposure assessment approaches. INTERCAMBIO is coordinating a diverse stakeholder community and developing a new health research agenda in interaction with other (European) initiations. INTERCAMBIO seeks to contribute to supporting decent green jobs.

(iii) **PROSPERH**. The PROSPERH (Promoting Positive Mental and Physical Health at Work in a Changing Environment) research project underscores the urgency of prioritizing holistic workplace well-being. With the evolving nature of work—marked by remote and hybrid arrangements, technological advancements, and increasing job demands—it is imperative to integrate strategies that enhance both mental and physical health in professional settings. PROSPERH enhances workplace health by providing evidence-based digital interventions, targeted at the individual, peer and organizational levels, that help prevent and manage work-related physical and mental health conditions and prepare workplaces for evolving environments. Employers will benefit from improved health promotion strategies, while policy-makers will gain valuable insights for shaping effective interventions that foster healthier behaviors and workspaces. The PROSPERH intervention is being delivered via the PROSPERH portal and application in 11 countries (Albania, Australia, Denmark, Germany, Hungary, Ireland, Kosovo, The Netherlands, Portugal, Spain and Turkey), targeting the construction and healthcare sectors and workers in telework and ICT-based mobile work settings.

(iv) **SONATA.** The SONATA project aims to augment currently existing and develop novel architectural adaptation technologies, including smart building systems that optimize indoor environmental quality and comfort (21) and robotically moveable acoustic walls and ceiling panels that reconfigure space (22). The project is assessing the medical, situational, and social impacts of these technologies across three shared workplace contexts: an open-plan office, a hybrid co-working space, and a set of home offices, in four different countries: Belgium, Germany, Italy, and The Netherlands. Accordingly, it will also establish a set of empirically grounded recommendations on how to (a) measure, compare, and optimize the health and well-being benefits of architectural adaptation technologies; (b) establish the ‘orchestration’ of multiple adaptive technologies in order to augment their joint benefit; and (c) prescribe the equitable distribution of these benefits among co-workers that share the same office. Ultimately, the SONATA research envisions a shared office workplace that is able to adapt to the ever-changing needs and preferences of individual workers, instead of expecting that these workers should constantly adapt to the rather generalized conditions of their workplace.

(v) **WAge.** WAge focuses on how physical and psychosocial workplace factors interact to influence health, well-being, and work sustainability across age groups. As working lives grow longer and workforce demographics shift (23), the project aims to support healthier and more inclusive workplaces by developing the WAge Index—a practical assessment and decision-support tool for organizations. The Index will help identify risk patterns and guide the design of tailored interventions that promote physical and mental health throughout the working life course. WAge combines mixed-methods field studies, participatory modelling, and cross-country collaboration in automotive and service sector workplaces in Spain, Portugal, and Poland, engaging workers, employers, and occupational health professionals. With an emphasis on age and gender-sensitive data, the project seeks to provide evidence and frameworks that are both scientifically robust and actionable in real-world occupational health contexts.

## Expected achievements

Together, the five projects in WISEWORK-C comprise a collaborative framework to support the creation of comprehensive, innovative solutions adaptable to a wide range of workplace environments. This framework advances the field by:

Bridging diverse domains of expertise, including mental, physical, and environmental health, as well as policy development, while maintaining a strong focus on real-world application. This transdisciplinary approach integrates evidence across disciplines to design holistic interventions that are both practical and scalable.

Addressing post-pandemic changes to the work environment. Due to the increased shift to remote and hybrid work, many organizations face new challenges, such as isolation, stress, and ergonomic issues from extended remote work.

Developing common data practices [eg, findable, accessible, interoperable, reusable (FAIR) principles, shared repositories] for robust, transparent research. By collaborating on data management and analysis, WISEWORK-C enhances the reproducibility and comparability of findings across countries and sectors—an important step for epidemiological rigor.

Through international and interdisciplinary collaboration, WISEWORK-C aims to contribute to a new state-of-the-art in sustainable, health-promoting workplaces. Its evidence-based findings will be made available to the scientific community and translated into innovative interventions that support workers’ physical and mental health. These insights will also inform policies and guidelines for public authorities, employers, organizations, and social partners. Looking ahead, the vision of the future workplace must respond not only to technological and demographic shifts but also to complex global challenges, including geopolitical instability, economic uncertainty, and the effects of climate change. WISEWORK-C envisions work environments that are not only digitally and ecologically advanced, but also socially responsive — capable of supporting diverse worker needs, mitigating emerging risks, and strengthening social cohesion in times of crisis. By identifying early warning signals, creating inclusive design strategies, and shaping evidence-based frameworks, the cluster supports a vision of work that is equitable, adaptive, and future-ready.
